# Development and validation of a 3D-printed dosimetry phantom for paediatric radiology

**DOI:** 10.1007/s13246-026-01698-3

**Published:** 2026-02-18

**Authors:** Jonathon Richard Stone, Pejman Rowshanfarzad, Adriano Polpo, Chris Williams, Rikki Nezich

**Affiliations:** 1 School of Physics, Mathematics and Computing, The University of Western Australia, Crawley, WA Australia; 2 Department of Medical Physics and Radiation Engineering, Canberra Health Services, Canberra, ACT Australia; 3 Centre for Advanced Technologies in Cancer Research (CATCR), Perth, WA Australia; 4 Department of Medical Technology and Physics, Sir Charles Gairdner Hospital, Nedlands, WA Australia; 5 Department of Medical Imaging, Perth Children’s Hospital, Nedlands, WA Australia

**Keywords:** Anthropomorphic phantom, Dosimetry, Paediatric radiology, 3D printing, PLA tissue equivalency, Monte Carlo validation

## Abstract

Radiation dosimetry is essential in the optimisation and justification of medical imaging procedures. However, the complexity of modern imaging equipment often surpasses the capabilities of standard dose calculation software, necessitating the use of commercially available dosimetry phantoms, which are often prohibitively expensive. This study aimed to develop a cost-effective, 3D-printed newborn-equivalent dosimetry phantom for measuring organ and whole-body effective doses. Several Polylactic Acid (PLA)-based filaments were investigated for tissue equivalency through Hounsfield-value analysis via micro-CT (40–70 kVp) and clinical CT (70–140 kVp) measurements. Standard PLA at 93% (ρ = 1.14 g/cm^3^) and 26% (ρ = 0.41 g/cm^3^) infill density was selected for soft tissue and lung, respectively, while StoneFil composite PLA (FormFutura) at 81% (ρ = 1.21 g/cm^3^) infill was chosen for bone. The phantom was modelled on a modified Cristy and Eckerman newborn design, with 21 sections generated using MATLAB and printed on a Bambu Lab X1 Carbon 3D printer. A total of 186 thermoluminescent dosimeter (TLD) capsules were embedded in the phantom, and TLD measurements from whole-body 60 kVp radiographs were compared with Monte Carlo (PCXMC 2.0) simulations for validation. The phantom demonstrated accurate dosimetry for the radiographic exposure, with average organ doses closely matching the simulated exposure, and the effective dose (ICRP 103) within 2% of the simulation. The phantom required 135 h to print, with a material cost of A$165. This study successfully developed and validated a cost-effective dosimetry phantom for paediatric radiography, with the potential to print larger phantoms for older children. Future work will explore the phantom’s application in other X-ray imaging modalities.

## Introduction

Ionising radiation from medical sources accounts for the largest portion of artificial radiation exposure to the population, with over 90% originating from medical sources [1]. Radiation dosimetry plays a critical role in justifying and optimising the radiation doses administered during radiological examinations, especially for radiosensitive populations such as children and infants. Patient dosimetry in radiology is often based on measurable dose quantities of the primary X-ray beam, such as the dose-area product (DAP) for radiography and the dose-length product (DLP) for computed tomography (CT) [2]. These patient-independent metrics enable easy dose comparisons across imaging protocols, aiding in patient dose optimisation. However, a key limitation of these quantities is the difficulty in using them to accurately determine individual organ and tissue absorbed doses or the effective dose. These dose values can instead be estimated using physical anthropomorphic phantoms or computational dose simulations.

While anatomically accurate voxel-based phantoms exist, many Monte Carlo simulation programs used in radiology physics departments continue to rely on stylised phantoms based on the Medical Internal Radiation Dose (MIRD) and Oak Ridge National Laboratory (ORNL) models from the 1980s due to their simplicity and computational efficiency [3]. For example, PCXMC 2.0 (STUK, Helsinki, Finland), employs a modified version of the Cristy and Eckerman [4] phantom from the ORNL phantom series, which is defined by parametric equations that model tissue, organ, and bone volumes [4, 5]. These equations contain different parameters for different patient sizes and ages, including newborn, 1-, 5-, 10-, 15-year-old, and adult phantoms. The model categorises tissues into three homogeneous types—soft tissue, lung, and bone—each with a defined chemical composition and density. The newborn model differs in composition and density for soft tissue and bone compared to the other phantoms, reflecting the increased red bone marrow and oxygen content in infants.

Simulations with Monte Carlo-based software for planar radiography, such as PCXMC 2.0, are generally preferred over physical phantom measurements due to their efficiency, lower labour requirements, and reasonable accuracy. Studies comparing dosimetric results from these simulations with dose measurements from various size physical anthropomorphic phantoms and newer hybrid computational phantoms have shown comparable results between modalities, albeit with some limitations [6–9]. In radiography, a key limitation is the design and anatomical differences between physical and computational phantoms, leading to different organ dose measurements, particularly for organs located at the edge of field.

Although standard dose calculation software is suitable for planar examinations, it often falls short in accurately modelling more complex imaging techniques such as cone-beam CT (CBCT), slot-scanning, and dual-energy X-ray absorptiometry (DEXA). This limitation has been highlighted in studies comparing dosimetric outcomes from simulation software to physical measurements taken in the Alderson Rando phantom (Radiology Support Devices, Inc., California, USA) and various age-specific ATOM phantoms (Sun Nuclear, Virginia, USA) [10–12]. Consequently, physical anthropomorphic phantoms are often required for accurate dose measurement and optimisation. However, commercial phantoms can be prohibitively expensive and may differ anatomically and compositionally from computational phantoms. This issue is particularly significant in paediatric radiology, where a range of phantom sizes is needed to represent diverse patient populations.

To address this issue, advancements in fused filament deposition (FFD), commonly known as 3D printing, offer a promising solution. FFD is an additive manufacturing technique that involves melting a spool of thermoplastic filament and extruding it through a small nozzle to form objects layer by layer. Its enhanced accessibility and customisability allow for the creation of anatomically accurate phantoms in various sizes, as well as the simulation of anatomical inhomogeneities, making it a useful tool for medical physicists [13, 14].

In radiology and radiotherapy, a material’s tissue equivalency is determined by its X-ray attenuation characteristics. Many studies have evaluated the radiological properties of common 3D printing filaments, identifying several that closely approximate human tissues. Most filaments used in 3D printing are organic polymers with effective atomic numbers and densities similar to water and soft tissue. Examples include nylon, polylactic acid (PLA), acrylonitrile butadiene styrene (ABS), and polyethylene (PE), which have been shown to adequately resemble the X-ray attenuation characteristics of muscle, adipose tissue, and other soft tissues [15, 16].

Among these materials, PLA is the most widely used due to its ease of printing and mechanical stability. Studies have demonstrated that when printed with a rectilinear infill pattern at densities above 80%, PLA exhibits X-ray attenuation characteristics similar to soft tissue across diagnostic beam energies [13–22]. Additionally, PLA has an effective atomic number (Z_eff_) of 7.25, closely matching water’s Z_eff_ of 7.42, further supporting its suitability as a tissue-equivalent material [23]. At lower infill densities (≤ 40%), PLA effectively resembles lung tissue due to its similar composition but reduced density [15, 17, 19, 20]. Bone-equivalency can be best achieved using plastic filaments doped with high-Z materials, such as metal or stone. StoneFil PLA (FormFutura BV, Nijmegen, Netherlands), a composite PLA-based filament containing 50% ground stone particles, has been shown to exhibit radiological properties similar to bone [13, 14, 16, 18, 20, 24]. Kozee et al. [13] further demonstrated that by adjusting print infill densities, StoneFil can accurately represent trabecular/cancellous, cortical, vertebral, and rib bone.

This study aims to design, construct, and validate a cost-effective paediatric dosimetry phantom, based on the revised newborn model by Cristy and Eckerman [4], utilising FFD technology. The phantom will be directly comparable to radiology dose simulation software such as PCXMC 2.0, CT-Expo (SASCRAD, Buchholz, Germany), and Radimetrics (Bayer AG, Leverkusen, Germany). This is to be achieved through three key objectives: selecting and calibrating of commercially available filaments to achieve tissue equivalency, designing and 3D printing the phantom, and validating the phantom’s dosimetry by comparing results to PCXMC 2.0 Monte Carlo simulation.

## Methods

### Characterisation of PLA-based filaments

Investigation of the tissue equivalency of PLA-based filaments was carried out in four main stages: Printing samples of candidate PLA filaments, Hounsfield unit analysis at high- and low-beam qualities, calculation of theoretical tissue attenuation values, and selection of filament infill densities which best match the theoretical tissues. A comprehensive flowchart of this project’s methodology can be found in Appendix A.

#### Candidate filament printing methodology

To characterise the X-ray attenuation properties of candidate materials, a disc-shaped phantom was 3D printed with interchangeable plugs of varying material compositions and infill densities. Based on recommendations by Kozee et al. and Ma et al. [13, 23], two PLA-based filaments were investigated for their tissue equivalency across a range of diagnostic X-ray beam qualities. Standard white 1.75 mm PLA filament (ρ = 1.24 g/cm^3^) was selected for newborn soft tissue and lung equivalency, and FormFutura’s 1.75 mm Granite StoneFil composite PLA filament (ρ = 1.70 g/cm^3^) purchased from AUSTiC was chosen for newborn bone equivalency.

**Fig. 1:**
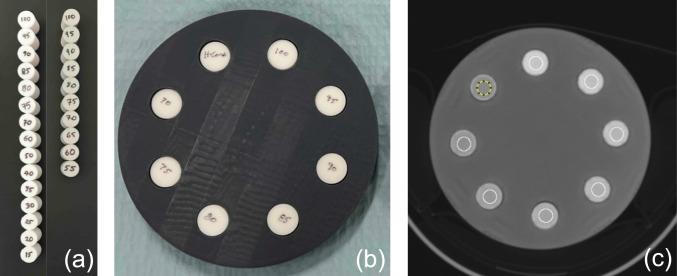
**a** Cylindrical white PLA (left) and StoneFil PLA (right) inserts with labelled infill densities. **b** PLA samples inserted into housing printed from standard grey PLA at 80% infill density. **c** CT slice of StoneFil samples with circular regions of interest for HU measurement

A series of cylindrical plugs, 18 mm in diameter and 20 mm in height were printed from both candidate materials over the range of infill densities shown in Fig. 1a (PLA: 15–100%, in 5% increments; StoneFil: 55–100%, in 5% increments). High- and low-density PLA samples were assessed for soft tissue and lung tissue equivalency, respectively, as further discussed in the following sections.

This study utilised the Bambu Lab X1 Carbon 3D printer (Firmware Version 1.06.05.01) (Bambu Lab, Shenzhen, China), fitted with an Automatic Material System (AMS) and a 0.4 mm stainless steel nozzle. Stereolithography (STL) files of the cylindrical samples were imported into Bambu Studio slicer software (Version 1.7.7.89) and printed using a 0.20 mm layer height, 220 °C nozzle temperature, and a 35 °C heat bed temperature. A standard rectilinear infill pattern with perpendicular infill direction between layers was used for all samples due to its simplicity and fast print speeds. Aside from infill density, all samples were printed with the default Bambu Studio settings for a 0.20 mm layer height and a 270 mm/s sparse infill print speed.

#### Attenuation of filaments at high beam qualities

The printed cylindrical plugs were inserted into several disc-shaped housings printed from standard PLA with an 80% rectilinear infill to approximate soft tissue (Fig. 1b). All samples were scanned in their housings using the Siemens SOMATOM Force clinical CT scanner (Siemens Healthineers, Erlangen, Germany) at 70–140 kVp, in 10 kVp increments, with a total equivalent filtration of 9.2 mm Al. All scans were performed at 400 mA, with a 1.0 mm slice thickness, and 1 s rotation time.

The resulting DICOM images were saved and analysed using ImageJ (Fiji version 2.14.0/1.54f), as shown in Fig. 1c. Regions of interest (ROIs) were generated within the cylindrical samples to measure the HU values for each infill density. To reduce statistical uncertainty, the mean HU of each sample was measured across 12 CT slices and averaged. Care was taken to avoid the walls of the samples, as well as the top and bottom surfaces, due to their higher density compared to the infill, which would have artificially increased the measured HU values.

As addressed below, this study employed two imaging modalities (clinical CT and micro-CT) to investigate the tissue equivalency of filaments at high and low beam qualities, respectively. To ensure accurate comparison between scanners with differing designs and filtration, beam quality was characterised using the half-value layer (HVL) rather than the nominal tube potential (kVp), as HVL provides a more representative measure for polyenergetic X-ray spectra.

Each scan energy (kVp) and respective HVL (mm Al) used on the Siemens SOMATOM Force scanner are presented in Table 1. HVLs were measured using the RaySafe X2 R/F dosimeter (Unfors RaySafe AB, Hovås, Sweden), positioned on the gantry bore directly opposite the X-ray tube and exposed during scout acquisitions (topograms). This set up enabled measurement of the effective HVL while avoiding errors introduced by bed movement or gantry rotation.

**Table 1:** Maximum beam energy (kVp) and corresponding HVL (mm Al) of Siemens SOMATOM Force CT with 9.2 mm Al equivalent total filtration, measured using the RaySafe X2 R/F sensor

Scan energy (kVp)	HVL (mm Al)
69.2	4.85
80.3	5.53
91.1	6.14
101.7	6.68
112.2	7.15
122.4	7.57
132.2	7.96
142.6	8.29

According to the manufacturer’s calibration certificate, kVp and HVL measurement accuracies are within 2% and 5%, respectively

#### Attenuation of filaments at low beam qualities

**Fig. 2:**
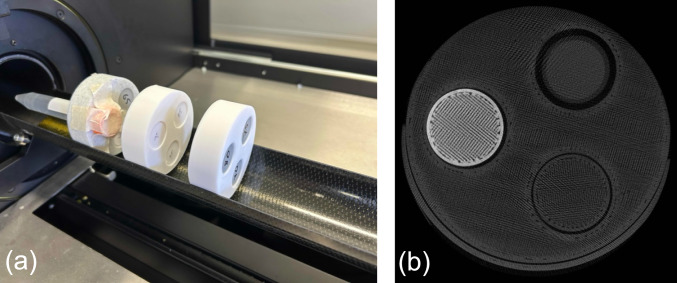
**a** Three 65 mm diameter cylindrical housings loaded into the Bruker Skyscan 1176 micro-CT with white PLA and StoneFil samples, and calibration water phantom. **b** Reconstructed micro-CT image of housing with StoneFil insert (left), white PLA insert (bottom right), and water phantom (top right)

To quantify the X-ray attenuation properties of the PLA samples at low beam qualities typical of planar radiography, this study used the preclinical Bruker Skyscan 1176 micro-CT (Bruker, Billerica, Massachusetts, USA). Due to the system’s limited 68 mm bore diameter, 65 mm disc-shaped housings with 80% infill were printed to hold up to three samples (Fig. 2a). Samples were scanned using 0.04 mm Cu + 0.5 mm Al added filtration, at beam energies ranging from 40–70 kVp, in 5 kVp increments, with measured half-value layers shown in Table 2. Like done on clinical CT, HVL measurements were taken with the RaySafe X2 R/F dosimeter during planar scout scans. All scans were performed at a 35 µm resolution, adjusting tube current and exposure time (Table 2) to optimise detector counts and minimise noise, as recommended in the Bruker Skyscan documentation. A water phantom of similar diameter to the printed samples was included in all scans for HU calibration.

**Table 2:** Scan settings for HU measurements using the Bruker Skyscan 1176 micro-CT with added Cu + Al filtration (2.1 mm Al equivalent). kVp and HVL were measured using the RaySafe X2 R/F dosimeter

Scan energy (kVp)	HVL (mm Al)	Tube current (µA)	Exposure time (ms)
40.0	1.33	600	325
45.2	1.41	450	325
50.9	1.56	375	300
56.2	1.73	350	250
61.8	1.89	275	250
66.9	2.04	350	150
71.8	2.17	250	200

Planar micro-CT image projections were tomographically reconstructed using Bruker NRecon software with 350 slices, 1/10 Gaussian smoothing, 0% beam hardening correction, and ring artefact reduction enabled. Greyscale values of the PLA samples were measured using Bruker CTan software by generating circular ROIs within the samples and averaging across all slices. Mean greyscale values of the measured samples were converted into HU numbers using the mean greyscale of the water phantom as a calibration reference.

Preliminary clinical CT HU results for lung-equivalent, low-density PLA showed minimal energy dependence (Fig. 7). As a result, low-density PLA samples were not scanned at lower beam qualities, as the findings were expected to be redundant.

#### Calculation of theoretical tissue attenuation values

The theoretical attenuation of newborn tissues, as defined by PCXMC, was calculated for all scanned energy points listed in Tables 1 and 2. X-ray spectra for each beam used to scan the samples were generated using the method developed by Boone and Siebert [25] with a tungsten anode, specified tube potential, and total filtration. The resulting spectra were output as photon histograms, binned in 1 keV increments, ranging from 0 keV to the maximum photon energy (equal to the tube potential).

The theoretical attenuation values for each compositional element of the tissues defined by PCXMC were derived from the mass attenuation coefficients presented in Chantler et al. (2000) and Chantler et al. (1995), taken from the National Institute of Standards and Technology (NIST) Standard Reference Database 66 [26]. As the energy increments given by the NIST did not match the 1 keV increments of the Boone and Siebert [25] spectra, linear interpolation was applied to the NIST mass-attenuation coefficient values to match them. The interpolated mass-attenuation coefficients of each of element (and water) were summed over the photon fluences of the spectrum at 1 keV increments, yielding the average mass-attenuation coefficient of each element for a particular spectrum (Eq. 1).

1 $${\left(\frac{\overline{\mu }}{\rho } \right)}_{Z,\hspace{0.17em} S} = {\sum }_{E = 0 keV}^{{E}_{max}}{\left(\frac{\mu }{\rho }\left(E\right)\right)}_{Z}\frac{{\Phi }_{S}^{free-air}\left(E\right)}{N}$$

Here ${\left(\frac{\mu }{\rho }\left(E\right) \right)}_{Z}$ represents the interpolated mass-attenuation coefficient of an element Z with energy E, ${\Phi }_{S}^{free-air}\left(E\right)$ is the in-air photon fluence for photons with energy E in a spectrum S, and N is the total number of photons in a spectrum S.

For each X-ray spectrum, the average mass-attenuation coefficients of the three newborn tissues were calculated by summing the average attenuation of the constituent elements, weighted by their mass composition. The linear-attenuation coefficients of the tissues were then determined by multiplying the mass-attenuation coefficients by their respective tissue densities (Eq. 2).

2 $${\mu }_{\text{tissue, S}}=\left[{\sum }_{Z}{\left(\frac{\overline{\mu }}{\rho }\right)}_{Z,S}{f}_{Z}\left({\mathrm{tissue}}\right)\right]{\rho }_{\mathrm{tissue}}$$

where ${\left(\frac{\overline{\mu }}{\rho }\right)}_{Z,S}$ is the average mass-attenuation coefficient of an element Z for a spectrum S, and ${f}_{Z}\left({\mathrm{tissue}}\right)$ is the compositional fraction of an element Z in a specific tissue.

Theoretical Hounsfield units for the PCXMC tissues were calculated using Eq. 3 for the tissue equivalency assessment of the printed samples analysed by clinical CT and micro-CT.

3 $$H{U}_{\text{tissue, S}}=\frac{{\mu }_{\text{tissue, S}}-{\mu }_{\text{water, S}}}{{\mu }_{\text{water, S}}}\times 1000$$

#### Optimal filament infill density selection

At each beam quality a linear regression model was fitted to the measured HU values against infill density for both standard PLA and StoneFil PLA. The linear models were used to determine the optimal filament infill densities required to best match the theoretically calculated HU values (Eqs. 1–3) of the PCXMC newborn tissues at each beam quality. As a result, each theoretical tissue was associated with a list of 15 optimal infill densities corresponding to the different beam qualities.

Weighted averages of the predicted infill densities for planar radiography and CT beam qualities were performed based on common kVps used in paediatric radiology. Since the phantom was intended to be utilised in both modalities, the final optimal infill density for each of the three main tissues was determined by averaging the infill percentage values separately calculated for radiography and CT beam qualities. For the uncertainty in the estimated optimal infill densities, we computed the predictive pointwise 95% confidence interval for each filament density at each half-value layer.

### Paediatric phantom design and construction

#### Computational phantom design

**Fig. 3:**
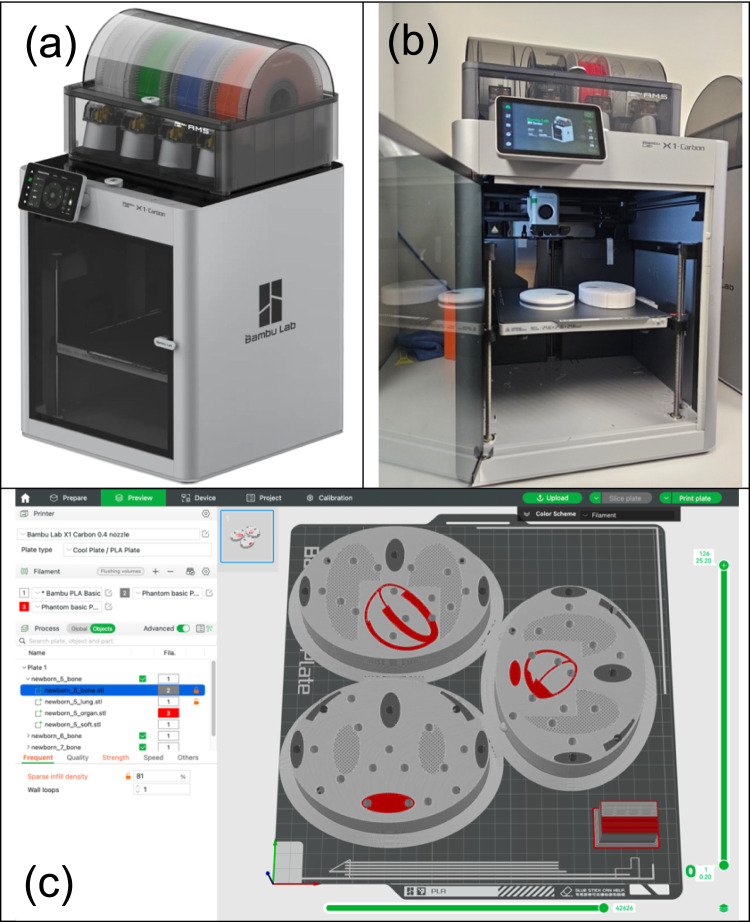
**a** Bambu Lab X1 Carbon 3D printer with Automatic Material System (AMS), taken from bambulab.com. **b** 3D printer set up with prototype printed slabs. **c** Screen capture from Bambu Studio of sliced slabs 5, 6, and 7 before printing, displaying organs, soft tissue, lungs, and bones

The parametric equations used to define the phantom regions were derived from Cristy and Eckerman [4] and implemented in a MATLAB script to computationally construct the phantom. The phantom was divided into twenty-one 25 mm slabs along the z-axis, with each slab containing 5.2 mm diameter holes to accommodate thermoluminescent dosimeter (TLD) capsules. The phantom was voxelised and rendered using an x–y resolution of 0.15 mm and a z resolution of 0.20 mm. Each voxel was assigned a corresponding tissue or material (air, lung, soft tissue, bone, and organ), based on its location, allowing for separate STL file exports for the different tissues (Fig. 3c and 9a). The phantom was composed of three major regions – head, trunk, and legs – the key dimensions of which are detailed in Table 3.

**Table 3:** Basic dimensions of the three major regions from this study’s modified Cristy and Eckerman [4] phantom

Body section	Max width (x)	Max depth (y)	Max height (z)
Head & neck	9.04 cm	11.54 cm	12.50 cm
Trunk	12.70 cm	9.80 cm	21.60 cm
Legs	12.70 cm	6.35 cm	16.80 cm

As briefly mentioned in the introduction, the revisions, including the addition of several organs, made to the design of the Cristy and Eckerman [4] phantom by PCXMC 2.0 are not documented. This study approximated these revisions using the parametric equations outlined in Appendix E1 of Deak et al. [27], along with additional approximations developed specifically for this work, as seen in Appendix B.

Additional 5.2 mm holes, aligned across slabs, were created to accommodate rods for phantom alignment. In total, 242 holes were distributed throughout the phantom, with 186 designated for TLDs. These TLDs covered 45 organs and bone regions, averaging approximately four per organ or bone. TLD hole locations were manually selected and assigned to specific organs to minimise organ dose sampling uncertainty. Some TLD holes spanned two organs; in these cases, each location was assigned both a primary and secondary organ designation.

#### Phantom 3D printing and construction

The STL files containing the 3D models of the separate tissues of the twenty-one phantom slabs were imported into Bambu Studio (software version 1.7.7.89) in preparation for 3D printing by the Bambu Lab X1 Carbon with AMS (Fig. 3c). For each slab, the STL files of each tissue type were jointly imported into Bambu Studio, where they were then separated and assigned their respective filament and infill density. Soft tissue and lung regions were printed using generic white PLA with a rectilinear infill density of 93% and 26% respectively, while bone regions were printed from StoneFil PLA with an 81% rectilinear infill density. These infill values were chosen based on the Hounsfield-value analysis detailed in the Optimal filament infill density selection section.

The phantom slabs were printed with the same default speed and volumetric flow settings as the cylindrical samples, with modifications including a single wall loop and the removal of 100% infill for the top and bottom layers. To enhance organ visualisation, the top and bottom surfaces of each slab included 0.20 mm thick STL files outlining organ shapes, printed with generic red PLA at a 93% infill. The red filament was limited to the superficial layers due to its potentially different attenuation properties compared to white PLA [23]. To minimise warping of the printed slabs, the auxiliary fan was disabled, the bed temperature was set to the maximum of 60 °C, and the nozzle temperature remained at 220 °C. The AMS enabled printing of generic PLA (for lung and soft tissue regions) and the StoneFil (for bone regions) on the same layer without the need for a dual extruder system. This is achieved by switching filaments at each layer—retracting the previous filament from the extruder and flushing the remaining material with the new filament. An AMS flushing volume of 150 mm^3^ was used for all prints to avoid filament mixing.

**Fig. 4:**
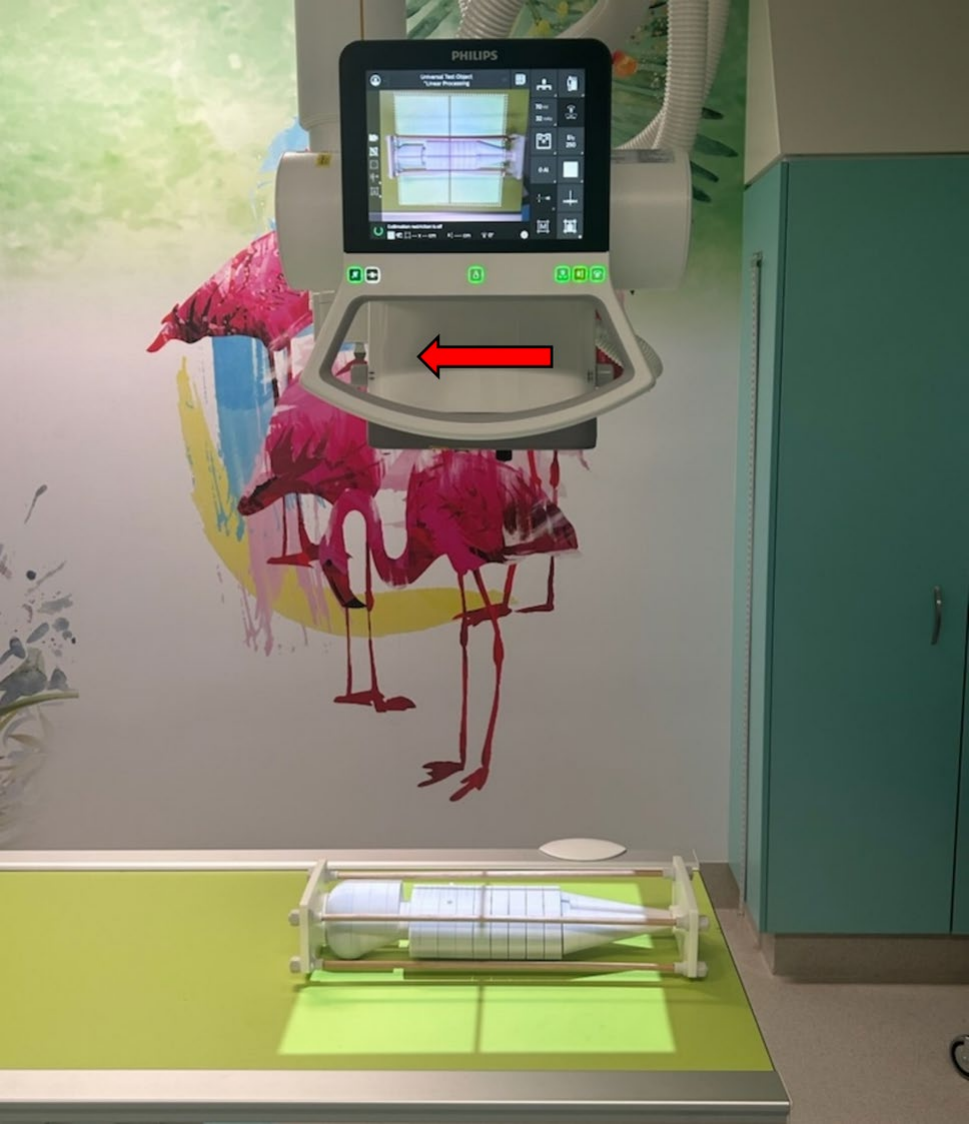
Photograph of the phantom during radiographic exposure on the Phillips Digital Diagnost C90. The red arrow indicates the cathode–anode direction, with the phantom’s head positioned on the anode side of the field

Additional components used in the phantom construction included alignment rods and a frame. The alignment rods, 5.2 mm diameter half-cylinders of varying lengths, depending on their position in the phantom, were printed with 93% infill white PLA for soft tissue equivalency. The frame was constructed from two plates with recesses for locating the head and the feet, printed with 25% infill white PLA, along with four wooden dowels fitted with M12 threads and nuts, printed from 50% infill Volcano PLA (FormFutura). The complete phantom, assembled in its frame, can be seen in Figs. 4 and 8b.

### Dosimetric validation

#### Radiographic exposure

This study utilised six batches of 50 low-dose LiF:Mg,Ti TLD capsules. Twelve TLDs per batch were designated as calibration rods across four dose points: 0 (background), 1, 10, and 20 mGy. At each dose point, three TLDs per batch were exposed alongside the RaySafe X2 R/F dosimeter for incident air kerma calibration. The dose-charge responses of the calibration rods from all TLD batches were modelled using a linear regression without an intercept, with correlation coefficients (R^2^) exceeding 0.9990 for all batches. Experimental TLD dose values were accompanied by 95% lower and upper prediction interval values.

The phantom, filled with TLDs, was exposed to a series of 60 kVp, 3.5 mm Al-filtered, 32 mAs radiographs on the Philips Digital Diagnost C90. For near-whole-body exposure, the beam was set to a 52 cm × 52 cm field size at a source-to-image distance (SID) of 110 cm and a 102 cm source-to-table distance (STD). The phantom was positioned with its z-axis aligned to the tube’s cathode–anode axis, with z = 12 cm at the beam centre (Fig. 4). The phantom’s head was positioned on the anode side of the X-ray tube, as commonly seen in paediatric anterior–posterior chest/abdomen exposures [28]. In this position, the phantom was exposed 20 times consecutively. 24 additional TLDs were placed on the phantom’s anterior and posterior surfaces (12 on the trunk, 4 on the head, and 8 on the legs) for skin dose measurements.

#### Monte Carlo simulations on PCXMC 2.0

The printed phantom's position during the 60 kVp radiographs was reproduced in PCXMC 2.0 (i.e., 52 cm × 52 cm field size at 110 cm SID, centred at z = 12 cm on the phantom). Similarly, the beam was modelled using 60 kVp, 3.5 mm Al total filtration, and a tube-anode angle of 13°. An incident air kerma of 25.81 mGy at the trunk was used to match the entrance dose observed in the physical exposure. The simulation was performed with 5 million histories, taking approximately 3 min to complete.

#### Thermoluminescent dosimeter analysis

This project employed a method based on the AAPM TG-61 protocol for kilovoltage X-ray beam dosimetry to convert the TLD incident air kerma measurements into tissue absorbed doses in the phantom [29]. The kerma to water was determined using the water-to-air mass-energy absorption coefficient (MEAC) ratio (Eq. 4). Given the low-energy photons in the 60 kVp spectrum, the dose to water was assumed to be equivalent to kerma as the range of secondary electrons is negligible [29]. Tissue doses were calculated using Eq. 5 and the tissue-to-water MEAC ratios provided in Table 4.

**Table 4:** Theoretically calculated mass-energy absorption coefficient ratios between PCXMC tissues, water, and air for a 60 kVp, 3.5 mm Al filtered spectrum. MEAC values were calculated using a method derived from Equation A.5 of AAPM TG-61 [29]. The full calculation method of the MEAC ratios is detailed in Appendix C

Media	Ratio value
Water, air	1.0178
Soft tissue, water	1.0108
Bone, water	3.0338
Lung, water	1.0405
Active bone marrow, water	0.8244

4 $${D}_{\mathrm{water}}\approx {K}_{\mathrm{water}}={K}_{\mathrm{air}}\cdot {\left[{\left(\frac{\overline{{\mu }_{en}}}{\rho }\right)}_{\mathrm{air}}^{\mathrm{water}}\right]}_{\mathrm{air}}$$

5 $${D}_{\mathrm{tissue}}={D}_{\mathrm{water}}\cdot {\left[{\left(\frac{\overline{{\mu }_{en}}}{\rho }\right)}_{\mathrm{water}}^{\mathrm{tissue}}\right]}_{\mathrm{air}}$$

The average absorbed doses for individual organs were calculated by averaging the tissue-converted TLD doses assigned to each organ. For composite organs such as active bone marrow, lymph nodes, skeleton, and colon, absorbed doses were calculated using a weighted sum based on weighting factors defined by Cristy and Eckerman and Tapiovaara et al. [4, 5]. The total body effective dose was calculated in accordance with the ICRP 103 method (Eq. 6).

6 $$E = \sum_{T}{w}_{T}{H}_{T}$$

where E is the total body effective dose, w_T_ is the ICRP 103 weighting factor of a given tissue/organ T, and H_T_ is the average absorbed dose to that tissue/organ T.

## Results

### Characterisation of PLA-based filaments

**Fig. 5:**
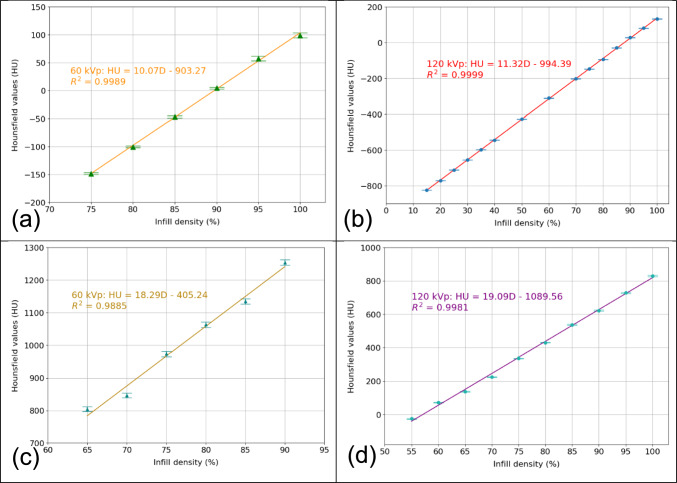
Representative subset of measured HU-infill response of standard white PLA **(a, b)** and StoneFil **(c, d)** samples for scans taken at 60 kVp by micro-CT (left column) and 120 kVp by clinical CT (right column). Micro-CT measurements were taken for a subset of both PLA and StoneFil samples. Linear regressions have been fitted between HU and infill, with equations and R^2^ values given. Error bars indicate the standard error of the mean (SEM) of HU measurements across the CT slices

Figure 5 displays the measured HU values for a representative subset of scanner kVp settings used – 60 kVp for micro-CT and 120 kVp for clinical CT – across the range of infill densities of the PLA and StoneFil samples. The results for both standard PLA and StoneFil samples illustrate a linear relationship between infill density and X-ray attenuation across several beam qualities, with R^2^ values for PLA samples exceeding 0.9990. Although the micro-CT measurements for the StoneFil samples do not display a correlation as strong as PLA’s and don’t meet the expected intercept of -1000 HU, their measured attenuation remains linearly dependent on infill density. Additionally, since the optimal infill densities were determined from both micro-CT and clinical CT results, these discrepancies have a reduced impact on the chosen phantom infill densities.

**Fig. 6:**
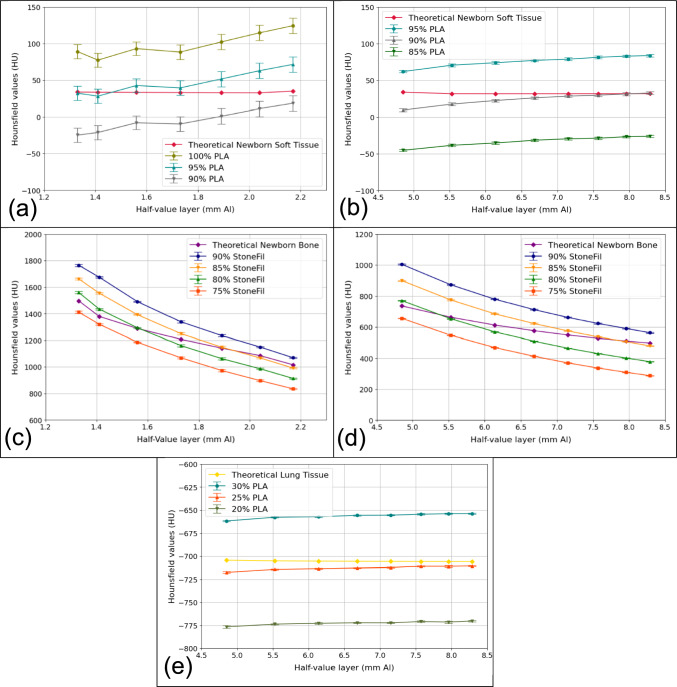
Measured Hounsfield values of soft-tissue equivalent white PLA **(a, b)**, lung equivalent low-density PLA **(e)**, and StoneFil **(c, d)** across micro-CT and clinical CT beam qualities. Theoretical HU numbers for PCXMC tissues have been plotted alongside measured values (clinical CT and micro-CT) for comparison of relative attenuation characteristics. Error bars indicate the SEM of HU measurements across the CT slices for each infill density sample, at each specific scan energy

Figure 6 compares the attenuation of the candidate filaments across the diagnostic beam quality range with the theoretically calculated PCXMC tissue HU values. The attenuation of PLA increases with half-value layer, stabilising at approximately 7.5 mm Al (120 kVp). This trend is expected as the photoelectric effect is more prominent at lower beam energies, and PLA's lower effective atomic number (Z_eff_ = 7.25) compared to water (Z_eff_ = 7.42) reduces its photoelectric absorption cross-section ($\propto \frac{{Z}^{3}}{{E}^{3}}$), lowering its relative linear attenuation and therefore HU at low beam qualities [23]. At higher beam qualities, where the Compton effect dominates, the attenuation becomes increasingly dependent on total electron density, leading to minimal energy dependence. Theoretically calculated attenuation values of newborn soft tissue exhibit minimal energy dependence due to its water-rich composition.

The attenuation of StoneFil and theoretical newborn bone follows an exponential decay-like trend with increasing beam quality, with StoneFil showing a steeper decay curve. This suggests that StoneFil's attenuation is more influenced by the photoelectric effect than newborn bone, likely due to its higher Z_eff_, resulting from calcium (Z = 20), silicon (Z = 14), and other high-Z elements present within the stone particles mixed into the filament. In comparison, newborn bone contains more oxygen due to its high red/active bone marrow content, reducing its Z_eff_ [17, 19].

The optimal infill densities and corresponding physical densities of PLA and StoneFil for each newborn tissue, calculated using the relative frequencies of X-ray energies encountered in our paediatric radiology department, are summarised in Table 5. These results indicate that the physical densities of high-density PLA and low-density PLA exceed those of soft tissue and lung respectively. This discrepancy arises from PLA’s lower photoelectric absorption cross-section, due to its lower Z_eff_, requiring a higher electron density to match the attenuation seen in the theoretical biological tissues. Conversely, the physical density of StoneFil is lower than that of newborn bone due to the higher Z_eff_, which requires a lower electron density.

**Table 5:** Optimal infill densities and corresponding mass densities of candidate filaments that best reproduce X-ray attenuation properties of the three infant tissue types defined by Cristy and Eckerman [4]

Tissue	Filament & Infill (%)	Printed mass density, ρ (g/cm^3^)	Cristy and Eckerman tissue density, ρ (g/cm^3^)
Soft	93% White PLA	1.143	1.04
Lung	26% White PLA	0.413	0.296
Bone	81% StoneFil	1.206	1.22

**Fig. 7:**
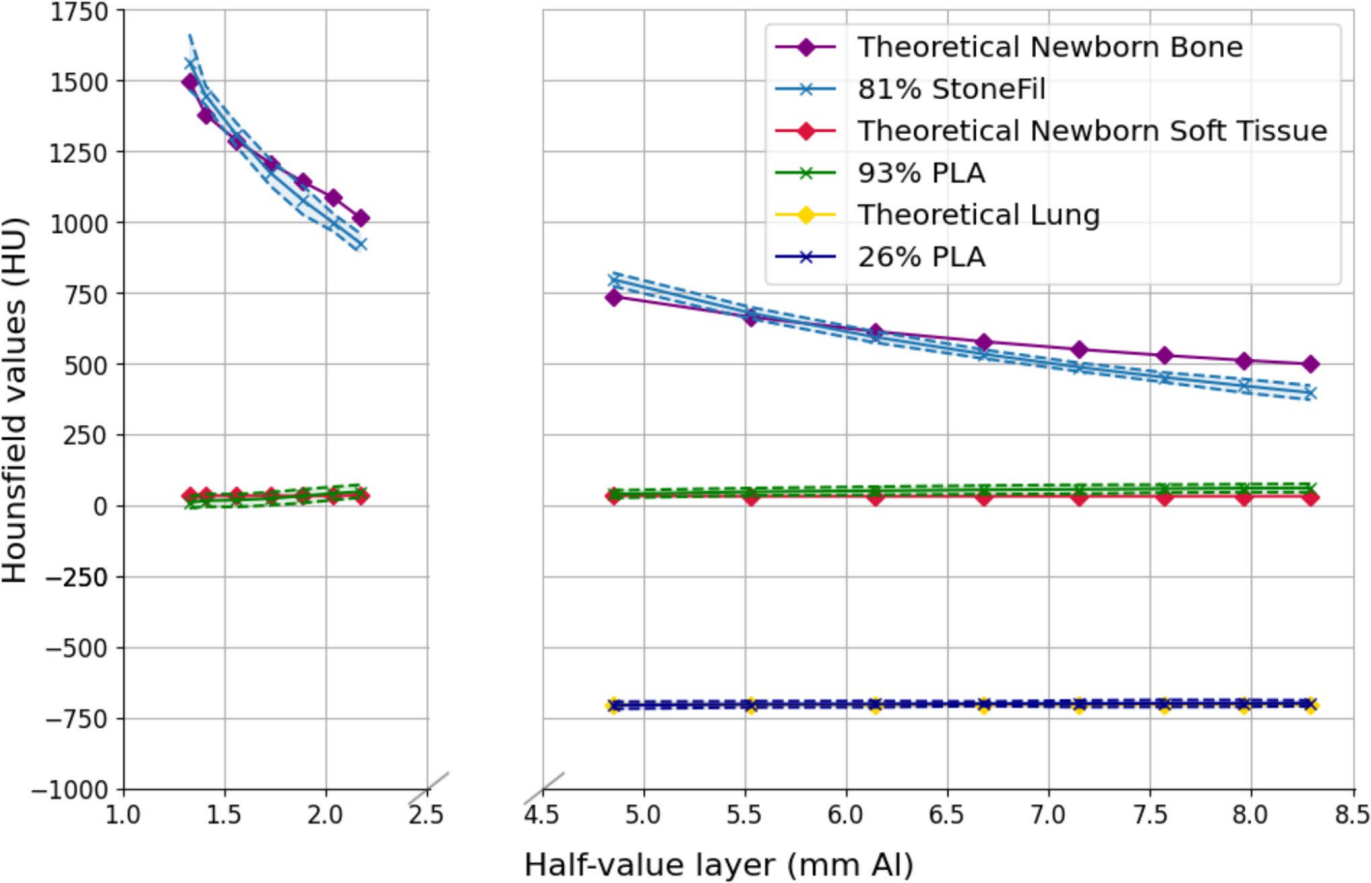
Predicted Hounsfield values of 3D printed materials at optimal infill densities for both micro-CT and clinical CT, plotted alongside theoretical HU values calculated for newborn soft tissue, bone and lung. The uncertainties in the infill density predictions are quantified using the 95% prediction interval, illustrated as the shaded area between the dashed lines surrounding each curve. Lung attenuation values are plotted only for the CT energy range

Figure 7 illustrates that the selected filaments and corresponding infill densities demonstrate suitable attenuation properties, effectively achieving tissue equivalency across the wide range of X-ray beam qualities encountered in diagnostic radiology. While the attenuation of the filaments does not precisely match that of the theoretical tissues, they represent a well-balanced compromise for the intended application.

### Paediatric phantom design and construction

**Fig. 8:**
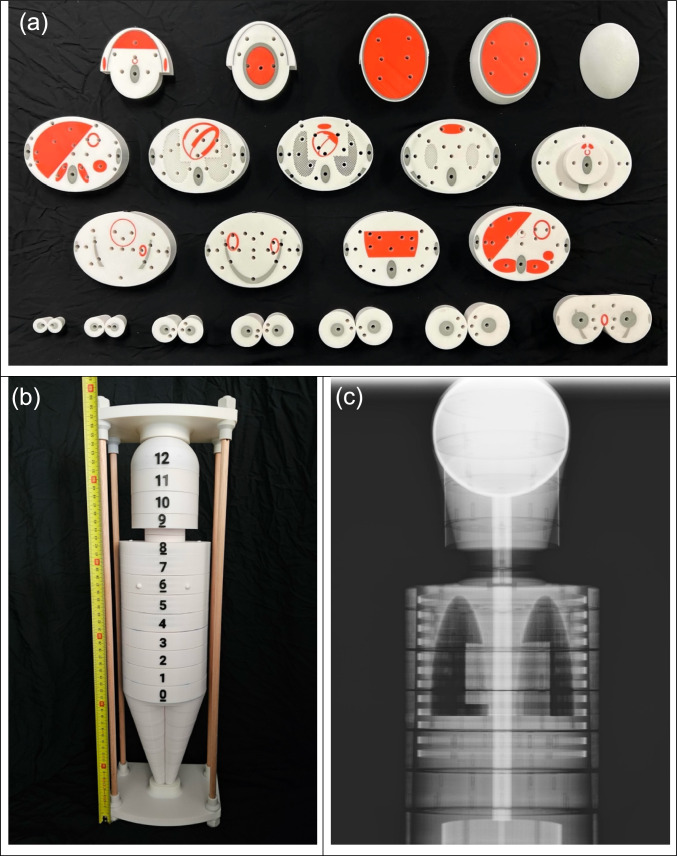
**a** Overhead photograph of the disassembled phantom with all 21 slabs shown. Bottom row (legs): Slabs -7 to -1; Second row (trunk): Slabs 0 to 3; Third row (trunk): Slabs 4 to 8; Top row (head and neck): Slabs 9 to 13. All slabs have had TLD capsules, plugs, and alignment rods removed. **b** Photograph of the fully assembled phantom in its frame. Trunk and head slabs have been labelled with their slab number on the anterior surface. **c** Radiographic image of the trunk and head region of the assembled phantom

The phantom was constructed using four 1 kg rolls of standard white PLA and one 500 g roll of Granite StoneFil PLA, resulting in a total cost of A$165 and 135 h of printing, excluding reprints and prototypes. Table 6 summarises the key attributes of the phantom, while Fig. 8 displays images of the phantom, disassembled and assembled in its frame.

**Table 6:** Key design properties of the paediatric phantom

Material cost	Print duration	Total mass	Basic dimensions
A$165	135 h	3.63 kg	9.8 × 12.7 × 50.9 cm

The printed phantom’s increased mass, excluding the frame, compared to the computational phantom (3.40 kg) can be attributed to the increased density of PLA required to achieve the same linear X-ray attenuation as the theoretical soft tissue, as previously discussed.

**Fig. 9:**
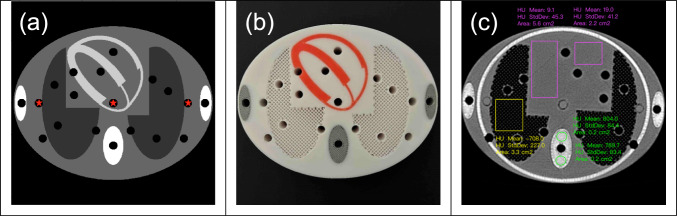
Computational, 3D-printed, and CT-scanned comparison of slab 5. **a** MATLAB Viewer window of slab 5, including TLD holes. The red stars indicate holes designated for alignment rods. Bony regions are shown in white, soft tissue in grey, lung in dark grey, organs (heart) in light grey, and air/holes in black. **b** Overhead view of 3D-printed slab 5. **c** CT scan of phantom slab 5 performed at 70 kVp on the Siemens Biograph Vision 600. Regions of interest measuring average HU and standard deviation have been generated in each tissue area (lung: -708, soft tissue: 14, bone: 796). All TLD holes were left empty except for holes used for alignment rods

A CT scan of the phantom was performed at 70 kVp (Fig. 9c), and DICOM images of its cross-sections were analysed to determine the Hounsfield units of the three primary tissue regions. As shown in Fig. 9c, the regions of interest within each printed tissue type exhibit HU values closely matching the theoretical values from the Cristy and Eckerman [4] phantom, highlighting the phantom’s success in achieving tissue equivalency.

### Dosimetric validation

**Fig. 10:**
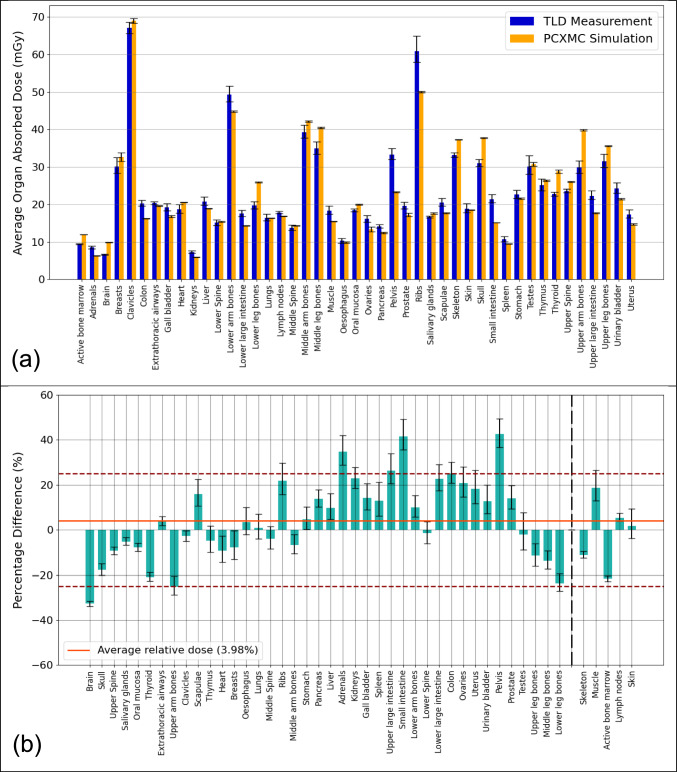
**a** Comparison of average organ doses: Measured vs. Simulated. Organs and tissues are listed in alphabetical order. Error bars on physical doses indicate the SEM of organ and tissue doses calculated from quadrature sum of individual TLD uncertainties. Error bars on simulated doses indicate the percentage statistical error returned in PCXMC. **b** Percentage difference between measured and simulated average organ absorbed doses. Organs have been rearranged from superior (left) to inferior (right). The skeleton, muscle, active bone marrow, lymph nodes, and skin are distributed throughout the phantom and have been separated on the graph by the vertical dashed line. Red horizontal dashed lines indicate ± 25% relative dose

Figure 10 compares the dosimetry results from the physical experiment with those from the PCXMC 2.0 Monte Carlo simulation. Relative dose differences calculated using Eq. 7, reveal strong agreement, with an average relative difference of 3.98%, and 39 out of 45 measured organ doses falling within 25% of the simulated results. A side-by-side organ dose comparison can additionally be seen in Table 10 of Appendix D.

7 $$\Delta = \frac{{D}_{TLD} - {D}_{PCXMC}}{{D}_{PCXMC}}\times 100\%$$

where ${D}_{TLD}$ and ${D}_{PCXMC}$ are the tissue/organ absorbed doses measured by TLDs and PCXMC 2.0, respectively.

Figure 10b demonstrates that the superior organs on the anode side received lower measured doses relative to the simulation, while inferior organs in the lower trunk received higher relative doses. This is likely due to the anode heel effect, which reduces photon intensity and increases mean photon energy on the anode side of the beam relative to the cathode side. While PCXMC 2.0 allows users to specify the tube-anode angle, it does not provide an option to define the tube orientation. Furthermore, the tube orientation is not mentioned in the documentation, suggesting that the tube-anode angle option in PCXMC 2.0 is used solely to generate the X-ray spectrum and does not account for the non-uniformity in the beam’s dose distribution caused by the anode heel effect.

PCXMC 2.0 simulation estimated a total body ICRP 103 effective dose of $E = 19.25 \pm 0.12$ mSv from the twenty exposures. Physical TLD dosimetry measured an effective dose of $E = {19.62}_{- 0.29}^{+0.40}$ mSv from the twenty radiographs, which is less than 2% higher than the PCXMC simulation result, further supporting the printed phantom’s dosimetric accuracy. This measurement corresponds to an average effective dose of $E= {0.981}_{- 0.015}^{+0.020}$ mSv per radiograph.

## Discussion

### Characterisation of PLA-based filaments

The tissue equivalency of commercially available 3D-printed materials has been widely studied in the context of both radiology and radiotherapy. While commercial filament characterisation has previously been completed at low beam qualities (i.e., below 70 kVp), like seen in Villani et al. and Ma et al. [22, 23], these were investigated by measuring linear attenuation coefficients using planar beam exposures. This study uniquely measured the Hounsfield units of PLA-based filaments across a comprehensive range of X-ray beam qualities, scanning at seven low-energies (40–70 kVp) on a preclinical micro-CT and eight high-energies (70–140 kVp) on a clinical CT. Previous studies, such as those by Kozee et al. and Ma et al. [13, 19] examined fewer energy points, using only five energies between 70 and 140 kVp. By including a broader range of energies, particularly for low beam qualities typical of radiography, this study enabled a more detailed analysis of the X-ray attenuation properties of candidate materials. As previously mentioned, while the micro-CT measurements of the StoneFil samples showed some anomalies, possibly introducing an additional uncertainty in the material’s characterisation, the results still offer valuable insight into the filament’s attenuation properties at low beam qualities.

Where other studies scanned 3D-printed filament samples in free air, this study involved samples housed in PLA with an 80% rectilinear infill density [13, 24]. In a similar approach, Ma et al. [18] embedded samples into a water phantom for consistency across several scans. Due to the relatively small size of the housing and the beam hardening correction algorithm applied during reconstruction, any effects of beam hardening on our HU measurements were likely minimised. Despite these methodological differences, the findings from this study remain consistent with previous research. As shown in Table 7, linear fits between HU and PLA infill density consistently feature intercepts near -1000—the expected HU value for air—across this and other studies. The slightly lower linear coefficients in Savi et al. and Okkalidis et al. [20, 24] may stem from differences in infill gradient settings between printers, as well as the lower kVp used by Okkalidis et al.

**Table 7:** Comparison of linear fits to HU values and rectilinear infill density of standard PLA with literature

Study	Linear equation	R^2^ value	Scan energy	Printer
This work	HU = 11.32D–994.39	0.9999	120 kVp	Bambu Lab X1C
Savi et al. [20]	HU = 9.98D–972	0.999	120 kVp	FlashForge Creator Pro
Okkalidis et al. [24]	HU = 10.636D–1065.3	0.9934	80 kVp	Mendel Prusa i2

This study observed increasing HU values for PLA samples with increasing scan energy. Similar trends have been reported in other studies, where carbon-based organic polymers such as nylon, ABS, and PLA exhibit increasing HU with beam energy [18, 19]. These trends are consistently attributed to the lower effective atomic number of these polymers compared to water and soft tissue, which are primarily composed of oxygen. Despite these differences, studies unanimously agree that PLA printed at high infill densities, typically between 80 and 95%, is adequately equivalent to soft tissue for the diagnostic X-ray energy range [13–18, 20–22]. This aligns with this study’s selection of 93% rectilinear PLA to represent infant soft tissue.

Theoretical lung tissue from the Cristy and Eckerman [4] phantom was found to be best approximated using PLA with a rectilinear infill of 26%. This finding agrees with the 30% infill density reported by Okkalidis et al. and Dancewicz et al. [24, 30]. In contrast, our results are not in complete agreement with Kunert et al. and Kairn et al. [16, 17] who report a 40% and 50% PLA infill density for lung, respectively. These differences stem from the different reference mass densities of lung tissue chosen between studies. For example, Kunert et al. [16] use 0.38 g/cm^3^ lung, compared to 0.296 g/cm^3^ lung used in this study, therefore requiring a higher infill density to match the increased attenuation.

Table 8 demonstrates that the observed attenuation characteristics of StoneFil PLA in CT beam energies are generally consistent with other similar studies by Kozee et al. and Ma et al. [13, 19]. Table 8 also highlights the shared agreement on StoneFil’s suitability for bone equivalence, despite differences in the reference bone densities and corresponding HU values used across studies. At 100% infill density, Kozee et al. [13] reported attenuation values of approximately 1200 HU at 70 kVp and 800 HU at 140 kVp, near the 1250 HU and 765 HU measured in this study. The higher attenuation at lower energies and lower attenuation at higher energies observed may stem from the differences in filtration or reconstruction techniques between CT scanners. Another source for this variation may have resulted from possible compositional differences in the StoneFil filament, like the discrepancies between different StoneFil batches noted in this study, discussed later under Limitations. Kozee et al. [13] additionally, observed that the energy dependence of StoneFil was closely aligned with bone-mimicking materials used in standard adult phantoms. While our results support this, it is less apparent when compared to Cristy and Eckerman [4]’s newborn bone, which displays a reduced energy dependence due to its lower density and effective atomic number.

**Table 8:** Comparison of filaments and infill densities selected for tissue equivalency with similar research

Study	Soft tissue	Lung	Bone*
This work	93% PLA	26% PLA	81% Granite StoneFil *(Infant homogenous* *528 HU @ 120 kVp)*
Kozee et al. [13]	84% PLA	25% Polyethylene	70% Granite StoneFil *(Vertebral cancellous* *277 HU @ 120 kVp)*
Ma et al. [19]	84.1% Nylon *(Adipose tissue* *-80 HU @ 120kVp)*	N/A^†^	73% StoneFil *(Average whole bone* *500 HU @ 120 kVp)*

*Reference bone types and their corresponding HU values at 120 kVp are included where applicable, due to variations in bone references across studies. ^†^Ma et al. (2022) did not investigate filaments for lung tissue equivalence

### Paediatric phantom design and construction

A key advantage of using fused-filament deposition 3D printing to construct anthropomorphic phantoms is the improved cost-effectiveness and customisability compared to traditional manufacturing methods. As seen throughout this study and supported by others, consumer-grade printers and filaments can replicate X-ray attenuation properties of human tissues across the diagnostic energy range while maintaining affordability. For example, the total cost of material used to construct the phantom in this study was A$165, in contrast to the A$38,000 purchase cost of the ATOM Newborn phantom, the closest equivalent commercial phantom. Even when considering the cost of the Bambu Lab X1 Carbon printer (A$2,219), the overall cost remains significantly lower. Furthermore, 3D printing enables the customisable design and fabrication of phantoms tailored to specific anatomical features or patient demographics. This adaptability allows for extensive modifications, accommodating for unique clinical scenarios that would be impractical or costly with traditional manufacturing techniques.

Many modern 3D printers offer fast print speeds with high accuracy, as demonstrated by the phantom’s total print time of 135 h over 10 days. This research utilised the X1 Carbon’s Automatic Material System to switch between PLA and StoneFil filaments for different tissue regions. Although this system ensured accurate transitions between tissue regions with minimal air gaps (Fig. 9c), print times were extended due to filament retraction and purging at each layer. Future improvements could involve using a dual extruder system to reduce filament waste and enhance efficiency. For instance, Kairn et al. [17] demonstrated high print qualities using a dual extruder system to simultaneously print soft tissue and bone regions with PLA and StoneFil respectively.

The use of commercial 3D printers and filaments makes this technology more accessible to a wider range of medical facilities, improving the ability to perform dosimetry in radiology. Designing the phantom in MATLAB provides improved customisability, with potential to generate 3D models of larger phantom sizes, such as the 1-, 5- and 10-year-old paediatric phantoms. This allows for a better coverage of the diverse patient sizes seen in paediatric radiology at a reduced cost.

### Dosimetric validation

Thermoluminescent dosimeter measurements and PCXMC 2.0 Monte Carlo simulation results of the radiographic exposures are in close agreement, with an average organ absorbed relative dose difference of 3.98%, and an ICRP 103 effective dose within 2% agreement. 39 out of 45 organs/tissue regions measured a dose within 25% of their simulated value, 10 of which were within 5% agreement. The largest differences in dose observed between the two methods were found in the small intestine, pelvis, adrenals, and brain, ranging from -33 to 43% (Table 10). As previously mentioned, a contributing factor to the discrepancies between measured and simulated dose values are thought to be caused by PCXMC 2.0 not considering the anode heel effect. The overall trend of the relative doses in this study aligns with the air kerma profile along the cathode–anode axis shown by Fig. 2 of Chan and Fung [31], where beam intensity is lower on the anode side than the cathode side. Variations in the relative doses are expected due to the discrete nature of the point dose measurements within the phantom. In contrast, PCXMC 2.0 calculates absorbed dose by averaging the total absorbed energy across an organ and dividing by its mass [5].

Several other sources of uncertainty include the intrinsic error of LiF:Mg,Ti TLDs, and the sampling uncertainty from the discrete TLD positions throughout the phantom. Several studies point out that low-dose LiF:Mg,Ti TLDs generally have a measurement uncertainty ranging from 3 to 10% [32, 33]. Although the air gaps between the phantom’s slabs were thin (approximately 1 mm wide), these air gaps aren’t seen in PCXMC 2.0, therefore they may have led to parts of the primary beam entering the phantom and introducing an added source of error in the TLD measurements. It should also be noted, however, that the TLD capsules position the sensitive volume of the LiF:Mg,Ti crystals towards the centre of each slab, partially minimising this effect. Further uncertainty in the average organ dose measurements is thought to have arisen from some experimental TLDs on the anterior side of the phantom being exposed to higher doses than the calibration rods, requiring extrapolation of the calibration curves.

When considering the potential dosimetric impacts of the infill, it is important to note that the infill pattern is diagonal along the x–y plane and alternates 90° between successive layers (Fig. 9b). This staggered material arrangement reduces the likelihood that certain beam lines travelling through the phantom have significantly different attenuation. Furthermore, the attenuation of different infill densities was characterised using CT (clinical and micro), where the beam is completely rotated around the sample, averaging the attenuation across all projection angles, therefore providing a measurement which is representative of the overall attenuation. However, this assumption is limited to beams travelling in the x–y plane. Due to the rectilinear nature of the infill pattern, air voids are created along the z-axis, particularly at lower infill densities like seen in the lung regions in Fig. 9b, which could lead to substantial differences in attenuation for beams with a caudocranial component. When the phantom is irradiated without a caudocranial beam angle, the dosimetric impact of these voids is expected to be minimal. This is evident by very close agreement (within 1%) observed in the measured and simulated lung doses, despite the lungs being printed with a lower infill density.

When directly comparing the results of this study’s phantom’s radiographic exposure to Ma et el. [9], who used the newborn ATOM phantom, several key similarities and differences in the phantoms’ dosimetries can be seen. Both phantoms exhibit total body ICRP 103 effective doses to within 4% of their respective procedures simulated in PCXMC 2.0. This illustrates that both phantoms display the potential for accurately calculating the total body effective dose. However, major differences between the two phantoms are seen in individual organ mean absorbed doses. While this study measured a maximum deviation of 42.6%, and most organs falling within 25% deviation (Fig. 10b), Ma et al. [9]’s ATOM phantom exhibited significantly more variation. Discrepancies ranged from -41% in the breasts to + 87.5% in the adrenals, with particularly notable overestimations for edge-of-beam organs, such as the thyroid and testes, where doses were over six times higher than the PCXMC simulated values. Although this study also found differences between measured and simulated doses for edge-of-beam organs, the discrepancies were much smaller, within approximately 30%. The significant deviations observed in Ma et al. [9] can be largely attributed to the differences in density, composition, and anatomy of the ATOM phantom compared to the Cristy and Eckerman [4] phantom, which were minimised in this study’s constructed phantom.

## Limitations

Although the composition of polylactic acid (PLA) is well-documented as it is an organic polymer, commercially available "standard" PLA filaments likely include unknown additives used for colouring or stabilising the filament. These additives may affect the attenuation properties of the filaments, particularly at lower X-ray energies. Ma et al. [18] highlighted that attenuation can vary between different filament colours, raising concerns about the experimental reproducibility when additives are present. Additionally, this study found inconsistencies in material density and attenuation characteristics across different batches of FormFutura’s Granite StoneFil filament. Users intending to use StoneFil for printing bone equivalent materials are advised to conduct their own attenuation characterisation, such as Hounsfield value analysis, to ensure accuracy.

Another limitation of PLA is its biodegradability, which can lead to deformation over time, particularly in warm, humid environments or with UV exposure [34, 35]. However, like other phantoms, it should be stored in a temperature-controlled environment with low UV and humidity. While no deformation has been observed in our phantom thus far, its long-term stability remains uncertain and may affect its suitability for extended use in medical dosimetry.

The method used to calculate the theoretical attenuation of computational phantom’s tissues is valid for the CT and micro-CT tube output but does not fully account for beam hardening caused by spectral changes as the beam is attenuated by either the PLA housing or the bowtie filter (clinical CT only). However, the relatively small diameter of the housing kept the inserted samples and corresponding regions-of-interest mostly within the beam centre, where the difference in bowtie filter thickness is expected to be small [36, 37]. Due to these mitigating factors, the overall impact of this limitation on the accuracy of the final infill densities is likely minimal, though difficult to quantify. This also applies for the CT images of the printed anthropomorphic phantom seen in Fig. 9c as its maximum deviation from the isocentre is approximately 6 cm. Similarly, the 60 kVp spectrum generated to calculate the tissue-to-water mass-energy-absorption coefficients (adapted from AAPM TG-61) does not account for spectral changes as the beam traverses the phantom. Consequently, small inaccuracies may have been introduced in absorbed dose calculations for tissues located deeper within the phantom.

## Future Work

Future studies could expand the phantom’s application to other imaging modalities, including cone-beam CT, dual-energy X-ray absorptiometry (DEXA), and slot-scanning systems. These dosimetric outcomes could be further validated by comparing the results with simulation software such as CT-Expo and Radimetrics. Additionally, the phantom presents an opportunity to evaluate the application of PCXMC 2.0 for more complex imaging methods.

This phantom is scalable to larger sizes, representing different age groups available in PCXMC, allowing for comprehensive validation across different patient demographics. Furthermore, incorporating radiochromic film into these phantoms, including the newborn model, could provide additional dosimetric verification. Although the phantom was primarily developed for dosimetry, there is opportunity for future validation of the phantom’s use for image quality assessments and obtaining exposure factors under automatic exposure control.

## Conclusion

This study successfully designed, built, and validated a cost-effective 3D-printed newborn dosimetry phantom for paediatric radiology, based on the Cristy and Eckerman [4] model. PLA-based filaments were thoroughly investigated for their X-ray attenuation characteristics, demonstrating that 93% PLA (ρ = 1.14 g/cm^3^), 81% StoneFil (ρ = 1.21 g/cm^3^), and 26% PLA (ρ = 0.41 g/cm^3^) infill densities provided equivalent properties for soft tissue, newborn bone, and lung, respectively, across the diagnostic X-ray energy range. The phantom’s 3D model, developed in MATLAB, ensured precise placement of thermoluminescent dosimeters for accurate dose measurements. Printed using the Bambu Lab X1 Carbon, the 21-slab phantom was produced over 135 h, costing approximately A$165. Absorbed dose measurements for a whole-body 60 kVp radiograph showed an average relative difference within 4% of PCXMC 2.0 simulation, confirming the phantom’s reliability in paediatric dosimetry. Its scalable design allows for adaptation for broader clinical applications, supporting the optimisation of radiological procedures.

## Figures and Tables

**Fig. 11:**
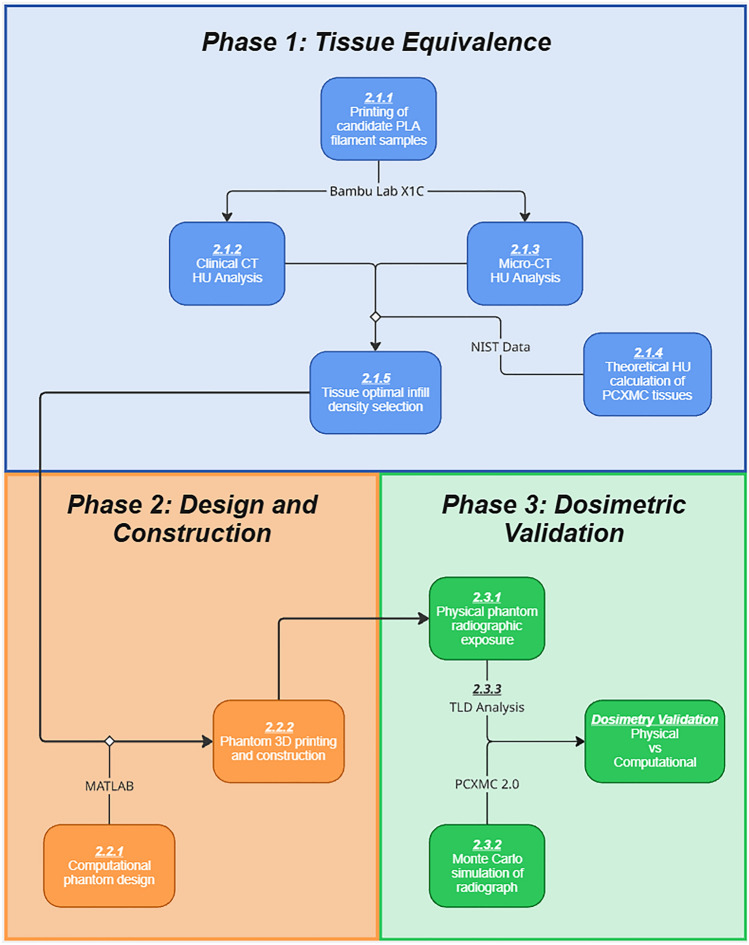
Flow chart representation of this study’s methodology. The methodology has been separated into three main phases: investigating tissue equivalency, phantom design and construction, and phantom dosimetric comparison

## Data Availability

The raw data and scripts used to support the findings in this study are available upon request via the corresponding authors.

## Appendix A: Methodology flow chart

See Fig. 11.

## Appendix B: Revised sections of the computational phantom

See Table 9.

**Table 9:** Parametric equations for revised or additional organs in the revised Cristy and Eckerman [4] phantom. Equations have been manually approximated from the PCXMC 2.0 viewer window. Equations derived from Appendix E1 of Deak et al. have been marked with an asterisk (*) [27]

Revision and description	Revised parametric equation(s)	Variables values
**Neck** Small circular cylinder superior of the trunk	${x}^{2}+{y}^{2}\le {r}_{n}^{2}$ ${C}_{T}\le z\le {C}_{n}$	${C}_{T}=21.6$ ${C}_{n}=23.3$ ${r}_{n}=2.80$
**Posterior of the Head** Half a right elliptical cone that connects the top of the neck to the base of the ellipsoid defining the top of the head	${\left(\frac{x}{{a}_{b}}\right)}^{2}+{\left(\frac{y-{y}_{1}}{b}\right)}^{2}\le 1$ $y\ge 0$ ${C}_{T}+{C}_{H1}\le z\le {C}_{n}$ ${a}_{b}=\left(\frac{{a}_{h}-{r}_{n}}{{C}_{T}+{C}_{H1}}\right)\cdot \left(z-{c}_{n}\right)+{r}_{n}$ ${b}_{b}=\left(\frac{{b}_{h}-{r}_{n}}{{C}_{T}+{C}_{H1}}\right)\cdot \left(z-{c}_{n}\right)+{r}_{n}$	${a}_{h}=4.52$ ${b}_{h}=5.78$ ${C}_{H1}=9.10$ ${C}_{T}=21.60$ ${C}_{n}=23.30$ ${r}_{n}=2.80$
**Prostate*** Sphere near the base of the trunk	${x}^{2}+{\left(y-{y}_{0}\right)}^{2}+{\left(z-{z}_{0}\right)}^{2}\le {r}^{2}$	$r=0.65$ ${y}_{0}=-1.37$ ${z}_{0}=0.66$
**Upper Spine** Repositioned into the neck	${\left(\frac{x}{a}\right)}^{2}+{\left(\frac{y-{y}_{1}}{b}\right)}^{2}\le 1$ ${z}_{3}\le z\le {z}_{4}$	$a=0.65$ $b=1.23$ ${y}_{1}=0.25$ ${z}_{3}=21.6$ ${z}_{4}=27.02$
**Oral Mucosa*** Portion of an elliptical cylinder medial of the facial skeleton	${\left(\frac{x}{a-d}\right)}^{2}+{\left(\frac{y}{b-d}\right)}^{2}\le 1$ $y\le {y}_{0}$ ${C}_{T}+{C}_{H0}+{z}_{1}\le z\le {C}_{T}+{C}_{H0}+{z}_{2}$	$a = 3.63$ $b = 5.43$ $d= 0.07$ ${y}_{0}=-3$ ${z}_{1}=2.76$ ${z}_{2}=4.56$
**Nasal Sinus*** Two spheres posterior to the facial skeleton	${\left(x\pm {x}_{0}\right)}^{2}+{\left(y-{y}_{0}\right)}^{2}+{\left(z-{z}_{0}\right)}^{2}\le {r}^{2}$	$r = 0.62$ ${x}_{0}=0.95$ ${y}_{0}=4.52$ ${z}_{0}=26.45$
**Extrathoracic Airways*** (Pharynx, Larynx, Trachea) The volume subtended by two concentric circular cylinders in the neck, anterior to the spine	${\left(\frac{x}{a}\right)}^{2}+{\left(\frac{y-{y}_{1}}{b}\right)}^{2}\le 1$ ${\left(\frac{x}{a-d}\right)}^{2}+{\left(\frac{y-{y}_{1}}{b-d}\right)}^{2}\ge 1$	$a = 0.38$ $b = 0.38$ $d = 0.12$ ${y}_{0}=-1.42$ ${z}_{1}=22.45$ ${z}_{2}=24.45$ ${z}_{3}=26.45$
**Parotid Glands*** Two ellipsoids lateral of the facial skeleton	${\left(\frac{x\pm {x}_{0}}{a}\right)}^{2}+{\left(\frac{y-{y}_{0}}{b}\right)}^{2}+{\left(\frac{z-{z}_{0}}{c}\right)}^{2}\le 1$	$a = 0.22$ $b = 0.88$ $c = 1.33$ ${x}_{0}=4$ ${y}_{0}=-1$ ${z}_{0}=25$
**Submandibular Glands*** Two ellipsoids near the base of the head	${\left(\frac{x\pm {x}_{0}}{a}\right)}^{2}+{\left(\frac{y-{y}_{0}}{b}\right)}^{2}+{\left(\frac{z-{z}_{0}}{c}\right)}^{2}\le 1$	$a = 0.44$ $b = 0.88$ $c = 0.44$ ${x}_{0}=2.68$ ${y}_{0}=-2.24$ ${z}_{0}=23.88$
**Sublingual Glands*** Two ellipsoids near the base of the head	${\left(\frac{x\pm {x}_{0}}{a}\right)}^{2}+{\left(\frac{y-{y}_{0}}{b}\right)}^{2}+{\left(\frac{z-{z}_{0}}{c}\right)}^{2}\le 1$	$a = 0.28$ $b = 0.88$ $c = 0.28$ ${x}_{0}=0.39$ ${y}_{0}=-3.86$ ${z}_{0}=24.26$
**Oesophagus** The volume subtended by two concentric circular cylinders from the base of the neck to the stomach	${\left(r-d\right)}^{2}\le {x}^{2}+{\left(y-{y}_{0}\right)}^{2}\le {r}^{2}$ ${z}_{0}\le z\le {z}_{1}$	${y}_{0}=0.75$ $r = 0.3$ $d = 0.12$ ${z}_{0}=10.80$ ${z}_{1}=21.60$
**Thyroid** Adjustment of previous thyroid parameters by Cristy and Eckerman [4] to be positioned more anterior and dilated laterally	Unchanged from Cristy and Eckerman [4]	$R = 0.87$ $r = 0.40$ $c = 1.42$ ${y}_{0}=-1.75$
**Facial Skeleton** Adjustment of the previous facial skeleton parameters to be narrower in the lateral direction to fit in the parotid glands	Unchanged from Cristy and Eckerman [4]	${a}_{1}=3.63$ ${b}_{1}=5.43$ $d = 0.07$ ${z}_{1}=2.16$ ${z}_{5}=8.18$

## Appendix C: Method for calculating phantom tissue-specific absorbed doses

To calculate the water-to-air and tissue-to-water Mass-Energy Absorption Coefficient (MEAC) ratios, as outlined in the AAPM TG-61, MEAC values of water and the constituent elements of the PCXMC tissues were sourced from the NIST Standard Reference Database 126 by Hubble and Seltzer [38]. A 60 kVp spectrum with 3.5 mm Al filtration was generated, and linear interpolation was used to align the elemental MEAC data with the spectrum’s energy increments. The interpolated MEAC values were summed over the spectrum’s photon fluences to determine the average MEAC for each element (Equation C.1), which was used to calculate the average MEAC for each tissue type in the phantom (Equation C.2).

C.1 $${\left(\frac{\overline{{\mu }_{en}}}{\rho } \right)}_{Z} = {\sum }_{E = 0 keV}^{{E}_{max}}{\left(\frac{{\mu }_{en}}{\rho }\left(E\right)\right)}_{Z}\frac{{\Phi }^{free-air}\left(E\right)}{N}$$

C.2 $${\left(\frac{\overline{{\mu }_{en}}}{\rho }\right)}_{tissue}={\sum }_{Z}{\left(\frac{\overline{{\mu }_{en}}}{\rho }\right)}_{Z}{f}_{Z}\left({\mathrm{tissue}}\right)$$

Equation C.1 is similar to Eq. 1 and is derived from Equation A.5 of TG-61. Here ${\left(\frac{\overline{{\mu }_{en}}}{\rho }\right)}_{Z}$ is the average mass-energy absorption coefficient for an element, Z, while all other terms remain consistent with those defined in Eq. 2.

## Appendix D: Measured vs. computational dose table

See Table 10.

**Table 10:** Comparison of average organ/tissue doses from physical measurement by TLDs and PCXMC 2.0 Monte Carlo simulation. Organs have been arranged in alphabetical order

Organ/Tissue	Average physically measured dose (mGy)	Average simulated dose (mGy)	Percentage difference (%)
Active bone marrow	${9.43}_{-0.17}^{+0.14}$	$12.04 \pm 0.01$	-21.68
Adrenals	${8.53}_{-0.46}^{+0.38}$	$6.33 \pm 0.10$	34.78
Brain	${6.67}_{-0.12}^{+0.12}$	$9.91 \pm 0.02$	-32.67
Breasts	${30.10}_{-2.42}^{+1.75}$	$32.64 \pm 1.18$	-7.78
Clavicles	${67.06}_{-1.64}^{+1.51}$	$69.00 \pm 0.62$	-2.82
Colon	${20.31}_{-0.86}^{+0.66}$	$16.26 \pm 0.08$	24.94
Extrathoracic airways	${20.42}_{-0.40}^{+0.37}$	$19.65 \pm 0.12$	3.94
Gall bladder	${19.22}_{-1.06}^{+0.90}$	$16.84 \pm 0.20$	14.18
Heart	${18.73}_{-1.33}^{+1.05}$	$20.63 \pm 0.08$	-9.23
Kidneys	${7.32}_{-0.29}^{+0.26}$	$5.95 \pm 0.05$	22.91
Liver	${20.79}_{-1.21}^{+0.95}$	$18.95 \pm 0.06$	9.70
Lower Spine	${15.16}_{-0.80}^{+0.71}$	$15.39 \pm 0.12$	-1.54
Lower arm bones	${49.30}_{-2.31}^{+1.92}$	$44.78 \pm 0.18$	10.09
Lower large intestine	${17.61}_{-0.93}^{+0.75}$	$14.36 \pm 0.11$	22.66
Lower leg bones	${19.78}_{-1.06}^{+0.91}$	$25.91 \pm 0.16$	-23.66
Lungs	${16.48}_{-1.01}^{+0.80}$	$16.35 \pm 0.05$	0.83
Lymph nodes	${17.85}_{-0.34}^{+0.27}$	$16.93 \pm 0.05$	5.43
Middle Spine	${13.75}_{-0.79}^{+0.64}$	$14.33 \pm 0.07$	-4.04
Middle arm bones	${39.28}_{-1.93}^{+1.62}$	$42.12 \pm 0.17$	-6.75
Middle leg bones	${34.88}_{-1.76}^{+1.49}$	$40.44 \pm 0.16$	-13.73
Muscle	${18.38}_{-1.22}^{+0.88}$	$15.49 \pm 0.02$	18.69
Oesophagus	${10.29}_{-0.66}^{+0.56}$	$9.95 \pm 0.18$	3.43
Oral mucosa	${18.50}_{-0.37}^{+0.34}$	$20.04 \pm 0.14$	-7.72
Ovaries	${16.15}_{-0.98}^{+0.82}$	$13.38 \pm 0.64$	20.71
Pancreas	${14.21}_{-0.51}^{+0.45}$	$12.49 \pm 0.21$	13.80
Pelvis	${33.31}_{-1.61}^{+1.35}$	$23.36 \pm 0.12$	42.58
Prostate	${19.70}_{-0.99}^{+0.79}$	$17.28 \pm 0.48$	14.02
Ribs	${60.93}_{-3.98}^{+3.02}$	$50.02 \pm 0.15$	21.82
Salivary glands	${16.71}_{-0.31}^{+0.29}$	$17.62 \pm 0.18$	-5.17
Scapulae	${20.54}_{-1.16}^{+0.93}$	$17.72 \pm 0.14$	15.90
Skeleton	${33.12}_{-0.61}^{+0.48}$	$37.28 \pm 0.04$	-11.15
Skin	${18.83}_{-1.42}^{+0.99}$	$18.53 \pm 0.04$	1.64
Skull	${31.01}_{-1.01}^{+0.88}$	$37.67 \pm 0.11$	-17.68
Small intestine	${21.48}_{-1.17}^{+0.90}$	$15.18 \pm 0.05$	41.50
Spleen	${10.74}_{-0.78}^{+0.64}$	$9.51 \pm 0.10$	12.97
Stomach	${22.63}_{-1.19}^{+0.96}$	$21.61 \pm 0.19$	4.74
Testes	${30.08}_{-2.98}^{+2.04}$	$30.74 \pm 0.52$	-2.15
Thymus	${25.09}_{-1.70}^{+1.35}$	$26.35 \pm 0.18$	-4.78
Thyroid	${22.77}_{-0.56}^{+0.52}$	$28.77 \pm 0.43$	-20.85
Upper Spine	${23.59}_{-0.45}^{+0.43}$	$26.01 \pm 0.16$	-9.32
Upper arm bones	${29.78}_{-1.84}^{+1.47}$	$39.80 \pm 0.24$	-25.18
Upper large intestine	${22.37}_{-1.34}^{+1.00}$	$17.71 \pm 0.11$	26.34
Upper leg bones	${31.48}_{-1.92}^{+1.64}$	$35.55 \pm 0.18$	-11.45
Urinary bladder	${24.21}_{-1.54}^{+1.17}$	$21.48 \pm 0.24$	12.70
Uterus	${17.41}_{-1.23}^{+0.98}$	$14.72 \pm 0.15$	18.26

## Appendix E: Phantom construction comparison—computational vs. physical vs. CT

Full comparison of computational (left), 3D-printed (centre), and CT scanned (right) phantom slabs. High resolution clinical CT images were taken at 70 kVp, 500 mA, and reconstructed using the Br62 kernel. All TLD plugs have been removed except for alignment rods. Slab 13 (top of the head) has not been included as it is a simple ellipsoid cover.

## References

[1] Pernicka F, McLean I (2007) Dosimetry in diagnostic radiology: an international code of practice. International Atomic Energy Agency, Vienna, Austria

[2] Bushberg JT, Boone JM (2011) The essential physics of medical imaging. Lippincott Williams & Wilkins

[3] Zaidi H, Xu XG (2007) Computational anthropomorphic models of the human anatomy: the path to realistic Monte Carlo modeling in radiological sciences. Annu Rev Biomed Eng 9(1):471–500. 10.1146/annurev.bioeng.9.060906.151934 17298237 10.1146/annurev.bioeng.9.060906.151934

[4] Cristy M, Eckerman K (1987) Specific absorbed fractions of energy at various ages from internal photon sources: 1, Methods. Oak Ridge National Laboratory (ORNL), Oak Ridge, TN (United States). 10.2172/6233735

[5] Tapiovaara M, Siiskonen T (2008) PCXMC, A Monte Carlo program for calculating patient doses in medical x-ray examinations. https://www.julkari.fi/bitstream/handle/10024/124342/stuk-a231.pdf?sequence=1 10.1093/jicru/ndi034 24170881

[6] Borrego D et al (2018) Assessment of PCXMC for patients with different body size in chest and abdominal x ray examinations: a Monte Carlo simulation study. Phys Med Biol 63(6):065015. 10.1088/1361-6560/aab13e 29465419 10.1088/1361-6560/aab13e PMC6376487

[7] De Oliveira PMC et al (2011) Assessment of organ absorbed doses in patients undergoing chest X-ray examinations by Monte Carlo based softwares and phantom dosimetry. Radiat Meas 46(12):2073–2076. 10.1016/j.radmeas.2011.06.058

[8] Golikov V et al (2018) A comparative study of organ doses assessment for patients undergoing conventional X-ray examinations: phantom experiments vs. calculations. Radiat Prot Dosimetry 178(2):223–234. 10.1093/rpd/ncx102 28981902 10.1093/rpd/ncx102

[9] Ma H, Elbakri IA, Reed M (2013) Estimation of organ and effective doses from newborn radiography of the chest and abdomen. Radiat Prot Dosimetry 156(2):160–167. 10.1093/rpd/nct050 23520199 10.1093/rpd/nct050

[10] Kim E-K et al (2018) Estimation of the effective dose of dental cone-beam computed tomography using personal computer-based Monte Carlo software. Imaging Sci Dent 48(1):21–30. 10.5624/isd.2018.48.1.21 29581946 10.5624/isd.2018.48.1.21 PMC5863016

[11] Piai A et al (2023) Assessment of PCXMC Monte Carlo simulations in slot-scanning-based examinations: comparison with in-phantom thermoluminescent dosimetry. Radiat Prot Dosimetry 199(3):277–289. 10.1093/rpd/ncac273 36583519 10.1093/rpd/ncac273 PMC9985171

[12] Wood T et al (2015) Validation of a technique for estimating organ doses for kilovoltage cone-beam CT of the prostate using the PCXMC 2.0 patient dose calculator. J Radiol Prot 35(1):153–163. 10.1088/0952-4746/35/1/153 25634880 10.1088/0952-4746/35/1/153

[13] Kozee M et al (2023) Methodology for computed tomography characterization of commercially available 3D printing materials for use in radiology/radiation oncology. J Appl Clin Med Phys 24(6):e13999. 10.1002/acm2.13999 37096305 10.1002/acm2.13999 PMC10243336

[14] Okkalidis N, Marinakis G (2020) Accurate replication of soft and bone tissues with 3D printing. Med Phys 47(5):2206–2211. 10.1002/mp.14100 32068889 10.1002/mp.14100

[15] Savi M et al (2021) Study on attenuation of 3D printing commercial filaments on standard X-ray beams for dosimetry and tissue equivalence. Radiat Phys Chem 182:109365. 10.1016/j.radphyschem.2021.109365

[16] Kunert P et al (2022) Tissue equivalence of 3D printing materials with respect to attenuation and absorption of X‐rays used for diagnostic and interventional imaging. Med Phys 49(12):7766–7778. 10.1002/mp.15987 36121424 10.1002/mp.15987

[17] Kairn T et al (2020) Quasi-simultaneous 3D printing of muscle-, lung-and bone-equivalent media: a proof-of-concept study. Phys Eng Sci Med 43:701–710. 10.1007/s13246-020-00864-5 32524450 10.1007/s13246-020-00864-5

[18] Ma X et al (2021) Classification of X-ray attenuation properties of additive manufacturing and 3D printing materials using computed tomography from 70 to 140 kVp. Front Bioeng Biotechnol 9:763960. 10.3389/fbioe.2021.763960 34912790 10.3389/fbioe.2021.763960 PMC8666890

[19] Ma X et al (2022) X-ray attenuation of bone, soft and adipose tissue in CT from 70 to 140 kV and comparison with 3D printable additive manufacturing materials. Sci Rep 12(1):14580. 10.1038/s41598-022-18741-4 36028638 10.1038/s41598-022-18741-4 PMC9418162

[20] Savi M, Andrade MA, Potiens MP (2020) Commercial filament testing for use in 3D printed phantoms. Radiat Phys Chem 174:108906. 10.1016/j.radphyschem.2020.108906

[21] Solc J, Vrba T, Burianova L (2018) Tissue-equivalence of 3D-printed plastics for medical phantoms in radiology. J Instrum 13(09):P09018–P09018. 10.1088/1748-0221/13/09/P09018

[22] Villani D, Rodrigues O Jr, Campos L (2020) Dosimetric characterization of 3D printed phantoms at different infill percentages for diagnostic X-ray energy range. Radiat Phys Chem 172:108728. 10.1016/j.radphyschem.2020.108728

[23] Ma D et al (2022) Mechanical and medical imaging properties of 3D‐printed materials as tissue equivalent materials. J Appl Clin Med Phys 23(2):e13495. 10.1002/acm2.13495 34878729 10.1002/acm2.13495 PMC8833282

[24] Okkalidis N, Chatzigeorgiou C, Okkalides D (2018) Assessment of 11 available materials with custom three-dimensional-printing patterns for the simulation of muscle, fat, and lung hounsfield units in patient-specific phantoms. J Eng Sci Med Diagn Therapy 1(1):011003. 10.1115/1.4038228

[25] Boone JM, Seibert JA (1997) An accurate method for computer‐generating tungsten anode X‐ray spectra from 30 to 140 kV. Med Phys 24(11):1661–1670. 10.1118/1.597953 9394272 10.1118/1.597953

[26] Chantler C, et al (2005) Detailed tabulation of atomic form factors, photoelectric absorption and scattering cross section, and mass attenuation coefficients for Z= 1–92 from E = 1–10 eV to E = 0.4–1.0 MeV (Version 2.1). [Online] Available: http://physics.nist.gov/ffast. Accessed 16 August 2023. National Institute of Standards and Technology, Gaithersburg, MD (United States). Ionizing Radiation Division. 10.18434/T4HS32. Originally published as Chantler, C.T. (2000), J. Phys. Chem. 29(4): p. 597–1056. 10.1063/1.1321055; and Chantler, C.T. (1995), J. Phys. Chem. 24: p. 71–643 10.1063/1.555974.

[27] Deak PD, Smal Y, Kalender WA (2010) Multisection CT protocols: sex-and age-specific conversion factors used to determine effective dose from dose-length product. Radiology 257(1):158–166. 10.1148/radiol.10100047 20851940 10.1148/radiol.10100047

[28] De Souza CD et al (2022) Protocol for reducing radiation exposure during pediatric thoracic radiography. J Med Imag Radiat Sci 53(3):437–443. 10.1016/j.jmir.2022.05.012 10.1016/j.jmir.2022.05.012 35750602

[29] Ma CM et al (2001) AAPM protocol for 40–300 kV X-ray beam dosimetry in radiotherapy and radiobiology. Med Phys 28(6):868–893. 10.1118/1.1374247 11439485 10.1118/1.1374247

[30] Dancewicz O et al (2017) Radiological properties of 3D printed materials in kilovoltage and megavoltage photon beams. Phys Med 38:111–118. 10.1016/j.ejmp.2017.05.051 28610691 10.1016/j.ejmp.2017.05.051

[31] Chan CTP, Fung KKL (2014) An investigation on the non-uniform distribution of radiation intensity output of diagnostic X-ray tubes. J Med Imaging Radiat Sci 45(3):223–229. 10.1016/j.jmir.2014.04.006 31051973 10.1016/j.jmir.2014.04.006

[32] Maia AF, Caldas LV (2010) Response of TL materials to diagnostic radiology X radiation beams. Appl Radiat Isot 68(4–5):780–783. 10.1016/j.apradiso.2010.01.002 20097569 10.1016/j.apradiso.2010.01.002

[33] Sadek A, Abdou N, Alazab HA (2022) Uncertainty of LiF thermoluminescence at low dose levels: experimental results. Appl Radiat Isot 185:110245. 10.1016/j.apradiso.2022.110245 35461124 10.1016/j.apradiso.2022.110245

[34] Hamad K et al (2015) Properties and medical applications of polylactic acid: a review. Express Polym Lett 9(5):435–455. 10.3144/expresspolymlett.2015.42

[35] Farah S, Anderson DG, Langer R (2016) Physical and mechanical properties of PLA, and their functions in widespread applications—A comprehensive review. Adv Drug Deliv Rev 107:367–392. 10.1016/j.addr.2016.06.012 27356150 10.1016/j.addr.2016.06.012

[36] Sommerville M, Poirier Y, Tambasco M (2015) A measurement‐based X‐ray source model characterization for CT dosimetry computations. J Appl Clin Med Phys 16(6):386–400. 10.1120/jacmp.v16i6.5231 26699546 10.1120/jacmp.v16i6.5231 PMC5691008

[37] Yang K et al (2017) Direct and fast measurement of CT beam filter profiles with simultaneous geometrical calibration. Med Phys 44(1):57–70. 10.1002/mp.12024 28102951 10.1002/mp.12024 PMC5543987

[38] Hubbell JH, Seltzer S (1995) Tables of X-ray mass attenuation coefficients and mass energy-absorption coefficients from 1 keV to 20 MeV for elements Z = 1 to 92 and 48 additional substances of dosimetric interest. National Institute of Standards and Technology, Gaithersburg, MD (United States). Ionizing Radiation Division. No. PB-95–220539/XAB; NISTIR-5632. [Online] Available: http://physics.nist.gov/PhysRefData/XrayMassCoef/cover.html. Accessed 31 July 2023. 10.18434/T4D01F

